# Simple and Effective Methods of Freezing Capercaillie (*Tetrao urogallus* L.) Semen

**DOI:** 10.1371/journal.pone.0116797

**Published:** 2015-01-23

**Authors:** Artur Kowalczyk, Ewa Łukaszewicz

**Affiliations:** Division of Poultry Breeding, Institute of Animal Breeding, Wrocław University of Environmental and Life Sciences, Wrocław, Poland; CNRS, FRANCE

## Abstract

A continuous decline in the number and range of capercaillie (*Tetrao urogallus* L.) in many European countries can be observed, mostly due to habitat destruction by human activity, unecological forestry management, and increased density of natural predators. *Ex situ in vitro* gene banks provide a unique opportunity to preserve the genetic material for future generations. Simple and effective cryopreservation methods for capercaillie semen are discussed. Semen was collected from seven males kept in the Capercaillie Breeding Centre at Forestry Wisła in Poland. Within five minutes after collection, ejaculates were diluted with EK diluent, then divided into two parts, and subjected to two freezing procedures: in pellets and in straws. In fresh semen, ejaculate clearness, viscosity, color and volume, as well as sperm concentration, motility and morphology, were evaluated, while in frozen-thawed semen only motility and morphology of sperm were determined. Fertilizing ability of thawed semen was examined for samples frozen in straws. Significant (P<0.05) differences between individual males were found in relation to the majority of fresh semen traits: ejaculate volume averaged 102.1 µL (varying from 49.0 to 205.0); average sperm concentration was 632.5 x10^6^ mL^-1^ (178.8–1257.1); percentage of live normal cells varied from 39.2 to 70.3% (58.7% on an average); percentage of motile cells ranged from 76.0 to 85.7%) and motility parameters were male dependent, as well. Both cryopreservation methods had a negative effect on morphology and motility of frozen-thawed semen; however, the straw method yielded 60.7% and the pellet method 42.5% of live cells in total in thawed semen (P<0.05), while the number of live normal (intact) cells was similar (22.4 and 22.2%, respectively). Egg fertility varied between 77.8 and 91.7% (average 84.4%). Both freezing procedures seem to be effective in obtaining acceptable viability and high fertilizing potency of thawed sperm and can be used to create a gene bank of capercaillie semen.

## Introduction

Semen preservation in domestic avian species is only rarely used in breeding practice, due mainly to economic reasons, despite its long-proven value for the optimization of male genetic potential [1], [2]. However, nowadays semen cryopreservation is the main method of storage of reproductive cells for the *ex situ* management of genetic diversity in birds [3]. Biotechnological methods used in poultry reproduction, such as artificial insemination (AI) or semen cryopreservation, are also adopted for a variety of free-living bird species, especially for endangered ones [4], [5]. The success of semen cryopreservation has already allowed the creation of semen banks of various wild avian species [5], [6], [7], [8].

In Poland, as in many other European countries, the capercaillie (*Tetrao urogallus* L.) is listed in the National Red Data Book [9]. According to the Decree of the Polish Ministry of the Environment of October 12, 2011 (“The protection of animal species”), among the actions aiming to protect endangered species are *ex situ in vivo* breeding centers, whose main objective is to obtain animals for further restitution or reintroduction. An increase in reproductive efficiency of capercaillies kept in a closed aviary system by the use of biotechnological methods contributes significantly to the achievement of this goal. *Ex situ in vitro* gene banks in the form of frozen semen provide a unique opportunity to preserve the genetic material and use it even after the death of the donor [6]. Furthermore, the use of AI in small populations, with diluted or cryopreserved semen, may allow preservation of biodiversity, without the need to transport the birds, which in such cases are exposed to severe stress [5].

The main critical points that affect cell structures and metabolism during freezing-thawing processes are interactions between sperm and cryoprotectant added to sperm, the temperature curve of cooling and freezing, as well as the type of cell packaging [3], [10]. Up to now, the creation of semen banks of endangered species has been performed using protocols developed for poultry breeds or lines [11]. Creating an *ex situ in vitro* sperm bank of wild animals and those kept in captivity (zoological gardens and breeding centers) requires cryopreservation methods that can be applied without access to sophisticated freezing equipment (e.g. a programmable freezer). Sperm cryopreservation in pellets is considered as one of them. It has been effective for freezing capercaillie semen, resulting in 80% fertility after AI with thawed semen [8]. The other simple method is sperm freezing in straws and in nitrogen vapor, without special freezing equipment [12], [13]. Straw freezing has the advantage first that semen is packaged, which is more hygienic; and second, the use of frozen semen from a cryobank requires strict identification of each sample. Pellets are not convenient in these conditions [14], [15].

The present study was therefore aimed at developing a simple and effective method for capercaillie semen cryopreservation in straws without access to sophisticated freezing equipment, as an alternative to the pellet method.

## Material and Methods

### Ethics statement

The National Forestry in Wisła District got permission (DOP-OZGIZ.6401.03.171.2011.km, dated on: May 10, 2011; expiry date: December 31, 2021) issued by the General Director of Environmental Protection, signed by dr. Michał Kiełsznia for keeping, reproduction and collection of the biological materials for experimental purposes, every year up to 50 adults Capercaillie (*Tetrao urogallus*) and 150 juvenile birds in the Capercaillie Breeding Center in Wisła Forestry District, Poland. The experiments carried out on capercaillie within Grant NN 311 081040 were approved by II Local Ethics Commission for Experiments Carried on Animals (Permit: NR 31/2010; issued on February 22, 2010) and both authors possess individual permission for carrying the experiments on animals, including capercaillie; Ewa Łukaszewicz—permit: Nr 23/2009, Artur Kowalczyk—permit: Nr 24/2009, both documents dated on June 19, 2009.

### Birds and their management

The experiments were carried out at the Capercaillie Breeding Centre belonging to Forestry Wisła District, Poland, in cooperation with Wrocław University of Environmental and Life Sciences. Seven *Tetrao urogallus* L. males between the ages of 2 and 10 year were used. During the reproductive season males were housed with or without females, in a separate roofed space (4.0 m x 7.0 m), under natural light and environmental conditions [16], [17]. All males had auditory and visual contact with females.

Semen collections were performed day by day or every second day, using the dorso-abdominal massage technique [17].

### Laboratory semen evaluation

Within five minutes following collection, each individual ejaculate was evaluated macroscopically (clearness, viscosity, color, volume) and microscopically (motility, sperm concentration and morphology). In the frozen-thawed semen the motility and morphology were examined.

Sperm motility was examined using a Sperm Class Analyzer (SCA, version 5.1, Microptic, Barcelona, Spain), a light microscope (Nikon Eclipse E200), with a x10 negative phase objective, a Basler camera (scA 780–54fc, Ahrensburg, Germany), a warm stage and a computer to analyze and save data. The following sets were used: medium VAP—50.0 µm s^-1^; low VAP—10 µm s^-1^; low LIN—50.0%. Before analysis, semen was diluted 1:10 in a warm (25°C) physiological solution (sodium chlorate 0.75%). Then 2 µL of the prepared sample was placed in a Leja 4 analysis chamber (Leja Products B.V., Holland) of thickness 20.0 µm. The slide was mounted on a stage warmer set at 38°C. The following motility parameters were included in this study: percentage of motile sperm, curvilinear velocity (VCL), straight-line velocity (VSL), path velocity (VAP), linearity (LIN), and amplitude of lateral head displacement (ALH). Minimum 500 cells were evaluated, and depending on sperm concentration, two to four analyses were performed per sample.

Sperm concentration was calculated with haemocytometer and Thoma-type grids. Sperm integrity and morphology were examined in nigrosine-eosin smears (300 sperms/slide) and assessed at 1250x magnification, under a light microscope (Nikon Eclipse E100). Sperm were morphologically categorized into six classes as described by Łukaszewicz et al. [17]; the results were expressed in percentages (300 cells = 100%).

For every male the semen quality factor (SQF) was calculated according to the following pattern: sperm concentration (n x10^6^ mL^-1^) x ejaculate volume (mL) x live normal sperm (%) /100%.

### Fertilizing ability test of thawed semen frozen in straws

The true sperm fertilizing potency (by female insemination and calculating hatching results) was examined for semen frozen in straws, exclusively. The efficiency of capercaillie semen frozen in pellets has already been tested in our previous study [8]. Moreover, such a procedure also arose from the limited number of females available for our experiment. Three females were left to our disposal. Since the number of progeny for further reintroduction is very important, in order to avoid the risk of getting the unfertile eggs from the inseminated females, we decided to use the semen of the “best male” (basing on motility and morphology of thawed sperm), which was not related to any of the inseminated females. It was male no. 51. Females were inseminated intravaginally, at a depth of about 2 cm, via the finger-guided method, using a plastic pipette, and with a dose of 100 µL of thawed semen. The first AI was performed 2–4 days prior to oviposition, the next two in two-week intervals. At every AI about 9 million live normal sperm were deposited. Laid eggs were naturally incubated by capercaillie females and the fertility rate was calculated on the basis of the hatched chicks and macroscopic analysis of the unhatched eggs.

### Semen treatment and freezing procedures

Fresh, individually collected ejaculates, after preliminary evaluation, were diluted three-fold (one part semen: two parts diluent) at room temperature with EK diluent, then divided into two parts, and further subjected to different freezing procedures: in pellets and in straws.

The composition of EK diluent was as follows: 0.14 g potassium citrate, 0.21g sodium dihydrogen phosphate, 0.98 g disodium hydrogen phosphate, 0.7 g glucose, 1.4 g sodium glutamate, 0.2 g D-fructose, 0.7 g inositol, 0.1 g polyvinylpyrrolidone (PVP), 0.02 g protamine sulfate per 100 ml of bi-distillate water; osmotic pressure was 385 mOsmol/kg (Semi-micro osmometer Type ML, KNAUER, Berlin, Germany) and pH—7.8 (pH Meter P731, Wrocław, Poland) [18]. All components had pure analytic quality and were supplied by POCH Gliwice Poland, Fluka Buchs Germany, or Sigma St. Louis Mo USA.

The pellet cryopreservation procedure adapted from Tselutin *et al.* [19] was the same as in our earlier experiment [8]. Diluted semen samples were stored for 15 min at temperature –8°C, then dimethyl-acetamide (DMA, SERVA, Heidelberg, Germany) was added to a final concentration of 6%, and after a further five minutes of equilibration the solution was pipetted and plunged drop-by-drop directly into liquid nitrogen. Frozen pellets were transferred and stored in LN_2_ (–196°C). Pellets were thawed in a 60°C water bath for 3 to 5 s, after 1 to 12 months of storage.

The procedure of semen sample preparation and temperature decrease rate during freezing by the straw method were adopted from Łukaszewicz [18], but the freezing procedure was simpler, i.e. without a computerized freezing unit [20]. Diluted samples were stored at +4°C for 15 min, then mixed with 6% (v:v) dimethyl-formamide (DMF, SERVA, Heidelberg, Germany) in the final concentration, respired into 0.15 mL plastic straws and equilibrated at +4°C for a further 5 min. Semen samples were frozen in LN_2_ vapor, using a small polystyrene container and stainless steel stand, on which straws with semen were placed. During freezing the straws lay 2 cm above the liquid nitrogen surface, which allowed us to obtain a temperature decrease rate of 40°C/min, from +4°C do –110°C. Freezing temperature was monitored by a sensor (Delta OHM HD 2108.1, Padua, Italy) placed in one of the straws. After reaching a temperature of –110°C, straws were plunged into liquid nitrogen. Semen samples were thawed for 4–5 s, in a 60°C water bath, after 1–12 months of storage.

### Statistical analysis

Data obtained were analyzed statistically with Analysis of Variance (ANOVA). Duncan’s Multiple Range Test (DMRT) was used to compare means where significant interactions were identified (Statistica, version 8.0, StatSoft, Inc., Kraków, Poland, sp. z o.o.).

## Results

### Characteristics of the fresh capercaillie semen

Significant (P<0.05) differences between individual males were found in relation to the majority of evaluated semen traits. Ejaculate volume averaged 102.1 µL, varying from 49.0 (♂ no. 9) to 205 µL (♂ no. 58); average sperm concentration was 632.5 x10^6^ mL^-1^, varying from 178.8 (♂ no. 79) to 1257.1 x10^6^ mL^-1^ (♂ no. 9) (Table 1). No significant differences between males in percentage of live sperm in total (94.6% on average) were observed, but a significant difference (P<0.05) in relation to live normal cells was observed. The lowest value for the latter sperm was 39.2% (♂ no. 72) and the highest was 70.3% (♂ no. 49). Among deformed capercaillie sperm, the bulb head (20.0% on average) and midpiece deformations (8.1%) were the most frequently observed. The percentage of bent neck sperm (4.3%), spermatids (0.5%) and cells with “other deformations” (2.7%) was lower, and differences (P<0.05) between individuals were noted. Ejaculate quality expressed as semen quality factor (SQF) averaged 35.7, with significant (P<0.05) variation, from 7.0 (♂ no. 79) to 61.0 (♂ no. 49), between capercaillie males (Table 1).

Sperm motility of fresh semen samples averaged 82.2%, and, similarly as in the case of sperm morphology, both percentage of motile cells and motility parameters were individual male dependent (P<0.05) (Table 2).

**Table 1:** Characteristics of the fresh semen collected individually from capercaillie and further subjected to freezing (means ± SD).

**Male’s number**	**No of obtained ejaculates**	**Semen volume [µL]**	**Sperm concentration [n x10^6^mL^-l^]**	**Morphological forms of sperm [%]**							SQF^*)^
				**Live in total**	**Live normal**	**Bulb head**	**Bent neck**	**Midpiece deformations**	**Spermatids**	**Other deformations**	
51	12	102.0^**c**^±26.0	884.6^**ab**^±412.2	95.3±2.9	58.7^**b**^±12.6	21.9±10.3	2.5^**b**^±0.8	9.9^**b**^±5.8	0.5^**b**^±0.4	1.7^**b**^±1.4	52.2^**ab**^±42.6
58	9	205.0^**a**^±41.0	371.4^**cd**^±205.4	95.0±2.7	61.2^**ab**^±10.2	20.2±5.5	3.5^**b**^±1.8	4.6^**c**^±1.3	0.0^**b**^±0.0	5.5^**a**^±2.8	42.8^**ab**^±22.8
9	8	49.0^**d**^±25.0	1257.1^**a**^±750.6	96.5±2.5	59.4^**b**^±11.6	22.4±12.3	2.4^**b**^±1.3	10.1^**b**^±3.2	0.6^**b**^±0.5	1.6^**b**^±1.0	32.0^**b**^±17.2
49	11	136.4^**b**^±49.0	619.1^**bc**^±306.5	95.1±4.2	70.3^**a**^±11.0	19.1±9.5	1.9^**b**^±1.4	3.0^**c**^±1.1	0.0^**b**^±0.0	0.6^**b**^±0.3	61.0^**a**^±37.4
67	8	65.7^**d**^±24.0	604.3^**bc**^±307.5	95.0±2.6	68.8^**ab**^±13.2	17.6±11.0	2.7^**b**^±0.7	4.6^**c**^±2.1	0.2^**b**^±0.1	1.2^**b**^±0.7	31.7^**b**^±18.5
72	12	73.0^**cd**^±22.0	452.0^**cd**^±220.8	95.3±2.0	39.2^**c**^±9.8	17.4±6.9	10.6^**a**^±2.3	18.8^**a**^±3.6	1.3^**a**^±0.6	7.9^**a**^±4.0	13.6^**c**^±7.4
79	9	75.0^**cd**^±31.0	178.8^**d**^±69.2	94.3±4.1	58.8^**b**^±8.4	23.8±10.0	5.2^**a**^±2.7	5.6^**c**^±2.2	0.4^**b**^±0.2	0.6^**b**^±0.2	7.0^**c**^±3.4
**Total/Average**	**69**	**102.1±56.9**	**632.5±518.9**	**94.6±5.5**	**58.7±15.0**	**20.0±9.4**	**4.3±3.4**	**8.1±6.2**	**0.5±0.7**	**2.7±3.7**	**35.7±29.4**

^*)^ SQF = Sperm Quality Factor—sperm concentration [n x10^6^ mL^-1^] x ejaculate volume [mL] x live normal sperm [%] / 100%;

^a,b,c,d^—means in columns followed by different superscripts differ significantly (P<0.05).

**Table 2:** Characteristics of sperm motility in the fresh semen collected individually from capercaillie and further subjected to freezing (means ± SD).

**Male’s number**	**No of obtained ejaculates**	**Motile sperm [%]**	**Motility parameters**				
			**VCL [µm s^-1^]**	**VSL [µm s^-1^]**	**VAP [µm s^-1^]**	**LIN [%]**	**ALH [µm]**
51	12	82.0^**ab**^±6.8	79.1^**c**^±8.5	33.6^**b**^±9.3	52.4^**c**^±9.7	53.5^**a**^±7.8	4.8^**ab**^±1.4
58	9	81.3^**ab**^±5.8	89.5^**abc**^±23.2	40.9^**a**^±7.5	57.2^**bc**^±7.4	47.7^**ab**^±2.9	3.9^**b**^±0.6
9	8	85.6^**a**^±5.5	95.0^**a**^±23.2	30.6^**b**^±4.4	54.4^**c**^±10.0	34.4^**d**^±9.4	4.9^**a**^±1.3
49	11	85.5^**a**^±6.9	100.6^**a**^±11.4	41.1^**a**^±8.8	65.0^**a**^±5.3	41.9^**bc**^±12.5	4.5^**ab**^±1.1
67	8	85.7^**a**^±5.3	91.6^**ab**^±13.6	31.3^**b**^±7.3	55.5^**c**^±7.1	33.3^**d**^±5.0	4.4^**ab**^±0.9
72	12	76.0^**b**^±7.0	81.7^**bc**^±5.3	33.8^**b**^±6.2	53.0^**c**^±3.9	42.7^**b**^±8.7	4.0^**b**^±0.6
79	9	79.4^**ab**^±10.2	100.6^**a**^±7.1	35.3^**ab**^±4,6	61.9^**ab**^±3.8	35.2^**cd**^±9.0	5.3^**a**^±0.8
**Total/ Average**	**69**	**82.2±7.2**	**91.1±14.6**	**35.3±7.7**	**57.2±7.9**	**41.6±10.5**	**4.5±1.0**

VCL curvilinear velocity; VSL—straight-line velocity; VAP—path velocity; LIN—linearity; ALH—amplitude of lateral head displacement;

^a,b,c,d^—means in columns followed by different superscripts differ significantly (P<0.05).

### Characteristics of the frozen-thawed semen depending on the freezing method

**Fig 1:**
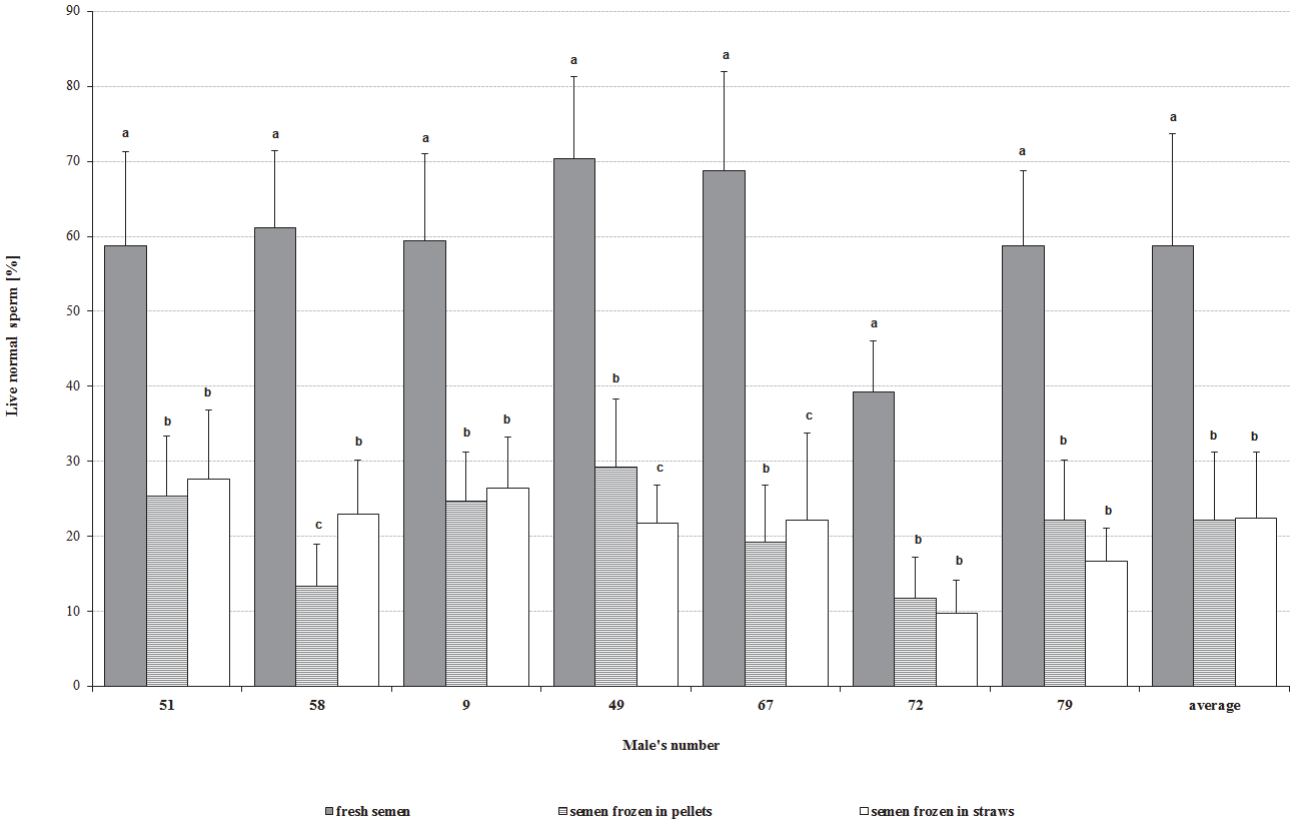
Comparison of the percentage of live normal sperm of individual capercaillie in the fresh and thawed semen samples, depending on cryopreservation method. ^a,b,c^ mean values of the percentage of live normal sperm in the fresh and thawed semen frozen by two different methods showed for one male and followed by different superscripts differ significantly (P<0.05).

**Fig 2:**
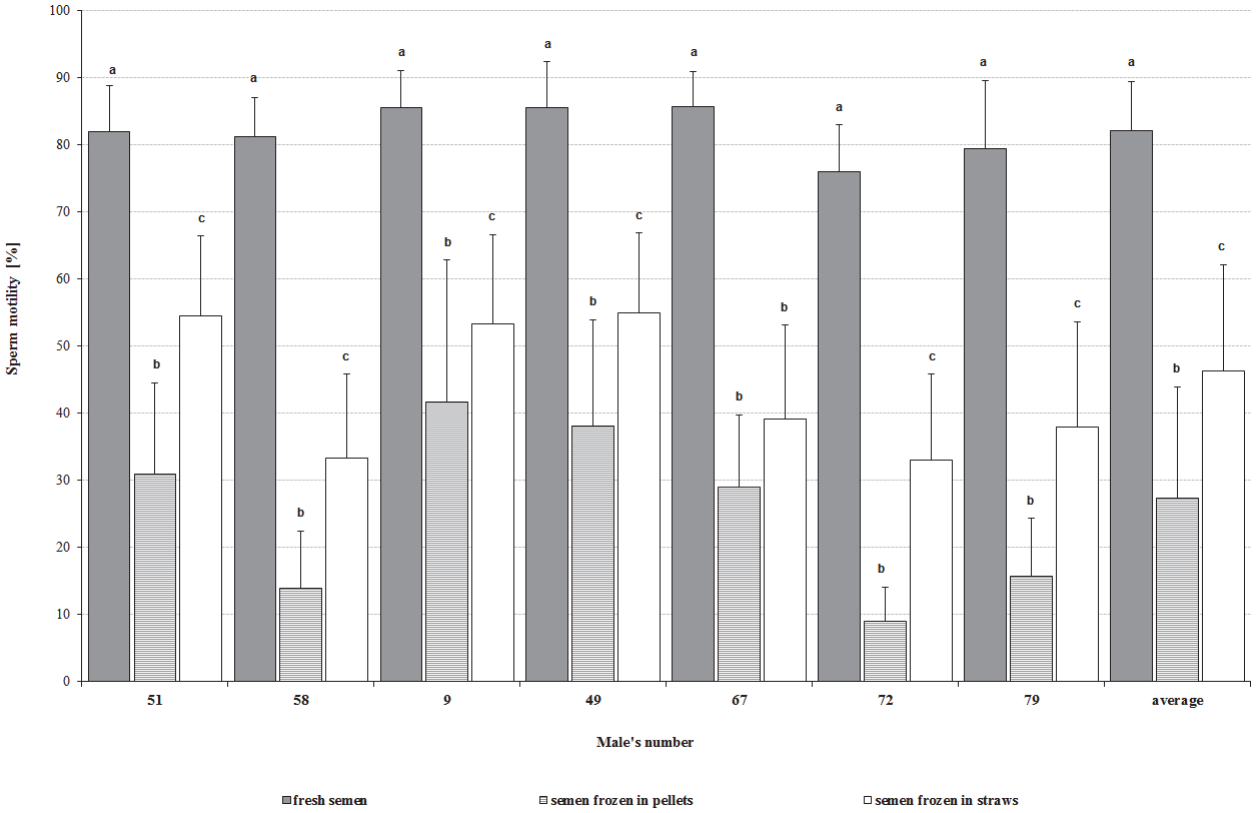
Comparison of sperm motility of individual capercaillie in the fresh and thawed semen samples, depending on cryopreservation method. ^a,b,c^ the mean values of sperm motility in the fresh and thawed semen frozen by two different methods showed for one male and followed by different superscripts differ significantly (P<0.05).

Compared to fresh semen, both applied cryopreservation methods (pellets and straws) caused a significant (P<0.05) decrease in percentage of live total (by 52.1% and 33.9%, respectively), and live normal (by 36.5% and 36.3%) sperm (Fig. 1). A significant (P<0.05) decrease in sperm motility by 54.8% of those frozen in pellets and by 35.9% of those frozen in straws (Fig. 2) as well as in motility parameters (VCL, VSL, VAP, ALH) was observed.

The average value of live sperm in total in semen frozen in pellets was significantly (P<0.05) lower, compared to the straw method, while the percentage of the most desired form, from the fertilizing potency viewpoint—the live normal cells (without evident morphological damage)—was similar. Moreover, in the straw method, a significantly (P<0.05) higher percentage of sperm with bulb head and bent neck could be observed (Table 3). The average motility of sperm cryopreserved in straw was higher (P<0.05) than of those frozen in pellets, but no differences in motility parameters were observed (Table 4).

**Table 3:** Characteristics of the frozen-thawed capercaillie semen depending on freezing method (means ± SD).

**Male’s number**	**Freezing methods**	**No of samples**	**Morphological forms of sperm [%]**					
			**Live in total**	**Live normal**	**Bulb head**	**Bent neck**	**Midpiece deformations**	**Other deformations**
51	pellets	12	43.8^**b**^±9.9 ^x^	25.4^**ab**^±8.0	8.3^**cd**^±5.3	6.8^**b**^±2.8	3.0^**a**^±1.8 ^x^	0.3^**ab**^±0.2
	straws		57.1^**abc**^±13.9 ^y^	27.6^**a**^±9.3	12.9^**c**^±5.1	9.6^**cd**^±4.5	6.1^**bc**^±2.6 ^y^	0.9^**b**^±0.4
58	pellets	9	38.4^**bc**^±5.7 ^x^	13.3^**cd**^±5.7 ^x^	9.1^**cd**^±2.9	13.6^**a**^±6.0 ^x^	2.0^**abc**^±1.3 ^x^	0.4^**ab**^±0.2
	straws		64.4^**ab**^±7.5 ^y^	22.9^**ab**^±7.3 ^y^	13.8^**c**^±5.7	21.3^**a**^±5.4 ^y^	5.4^**bc**^±4.0 ^y^	1.0^**b**^±0.9
9	pellets	8	39.7^**b**^±7.6 ^x^	24.7^**ab**^±6.6	7.3^**d**^±3.3 ^x^	4.8^**bc**^±3.0 ^x^	2.6^**ab**^±1.1 ^x^	0.3^**ab**^±0.1
	straws		68.2^**a**^±8.9 ^y^	26.4^**a**^±6.9	17.3^**bc**^±3.0 ^y^	13.3^**cd**^±6.4 ^y^	10.9^**a**^±3.4 ^y^	0.3^**bc**^±0.2
49	pellets	11	55.0^**a**^±16.3 ^x^	29.3^**a**^±9.0 ^x^	19.6^**a**^±7.6 ^x^	4.2^**bc**^±3.5 ^x^	1.7^**abc**^±1.0 ^x^	0.2^**b**^±0.3
	straws		66.4^**ab**^±13.3 ^y^	21.7^**ab**^±5.1 ^y^	24.0^**a**^±6.4 ^y^	14.7^**bc**^±9.0 ^y^	4.0^**c**^±2.4 ^y^	0.0^**c**^±0.0
67	pellets	8	40.8^**b**^±11.7 ^x^	19.2^**bc**^±7.6	16.7^**ab**^±3.9	3.7^**bc**^±2.0 ^x^	1.2^**c**^±0.7 ^x^	0.0^**b**^±0.0
	straws		56.1^**b**^±20.2 ^y^	22.1^**ab**^±11.7	17.7^**bc**^±9.2	9.1^**d**^±6.1 ^y^	6.9^**b**^±3.2 ^y^	0.3^**bc**^±0.1
72	pellets	12	30.0^**c**^±7.3 ^x^	11.8^**d**^±5.4	13.0^**bc**^±7.3	1.9^**c**^±1.4 ^x^	2.5^**ab**^±1.6 ^x^	0.8^**a**^±1.0 ^x^
	straws		62.2^**ab**^±13.3 ^y^	9.8^**c**^±4.3	14.6^**c**^±4.3	19.7^**ab**^±7.3 ^y^	11.4^**a**^±3.4 ^y^	6.7^**a**^±4.8 ^y^
79	pellets	9	41.5^**b**^±6.7	22.2^**b**^±8.0	11.1^**cd**^±4.6	6.5^**b**^±3.5	1.5^**bc**^±0.7 ^x^	0.2^**b**^±0.1
	straws		49.7^**c**^±10.7	16.7^**b**^±4.4	19.9^**b**^±6.0	8.4^**d**^±5.3	4.7^**bc**^±2.9 ^y^	0.0^**c**^±0.0
**Total/Average**	pellets	**69**	**42.5±12.4 ^x^**	**22.2±9.0**	**12.4±6.7 ^x^**	**5.6±4.7 ^x^**	**2.1±1.4 ^x^**	**0.3±0.5**
	straws		**60.7±13.8 ^y^**	**22.4±8.8**	**17.0±7.0 ^y^**	**13.6±7.9 ^y^**	**6.6±3.8 ^y^**	**1.0±2.2**

^a,b,c,d^—means in columns for the same freezing method, followed by different superscripts differ significantly (P<0.05);

^x,y^—means in columns for the same male indicate significant differences between freezing method (P<0.05).

**Table 4:** Characteristics of sperm motility in the frozen-thawed capercaillie semen depending on freezing method (means ± SD).

**Male’s number**	**Freezing methods**	**No of samples**	**Motile sperm [%]**	**Motility parameters**				
				**VCL [µm s^-1^]**	**VSL [µm s^-1^]**	**VAP [µm s^-1^]**	**LIN [%]**	**ALH [µm]**
51	pellets	12	31.0^**ab**^±13.5 ^x^	44.3^**bc**^±6.5	15.3^c^±1.3 ^x^	25.4^b^±3.8	34.9^**bcd**^±2.4 ^x^	3.5^**a**^±1.4 ^x^
	straws		54.5^**a**^±11.9 ^y^	39.7^**c**^±5.5	20.3^**ab**^±5.4 ^y^	27.8^**ab**^±4.8	51.5^**a**^±13.1 ^y^	2.5^**a**^±0.5 ^y^
58	pellets	9	13.9^**c**^±8.6 ^x^	41.0^**cd**^±3.9	17.4^**b**^±2.6	26.4^**b**^±3.3	40.1^**b**^±6.3	2.1^**cd**^±0.3
	straws		33.3^**b**^±12.5 ^y^	40.6^**c**^±2.2	18.7^**bc**^±4.0	26.3^**abc**^±2.4	44.0^**b**^±9.7	2.6^**a**^±1.0
9	pellets	8	41.7^**a**^±21.2 ^x^	53.2^**a**^±10.7 ^x^	19.6^**a**^±3.2 ^x^	31.9^**a**^±5.1 ^x^	39.1^**bc**^±11.2	2.9^**b**^±0.7 ^x^
	straws		53.3^**a**^±13.3 ^y^	42.1^**bc**^±4.3 ^y^	16.2^**cd**^±1.3 ^y^	25.5^**bc**^±2.8 ^y^	37.8^**c**^±8.2	2.3^**ab**^±0.4 ^y^
49	pellets	11	38.1^**ab**^±15.8 ^x^	45.4^**bc**^±5.9	14.5^**c**^±1.2	25.6^**b**^±2.8	33.9^**cd**^±3.2	2.7^**b**^±0.4
	straws		55.0^**a**^±11.9 ^y^	44.5^**b**^±5.5	14.7^**d**^±1.3	25.2^**bc**^±3.2	34.7^**c**^±2.4	2.6^**a**^±0.4
67	pellets	8	29.0^**b**^±10.8	47.7^**b**^±3.8	16.0^**bc**^±1.0	27.0^**b**^±1.5	36.2^**bcd**^±4.9	2.7^**b**^±0.2
	straws		39.2^**b**^±14.0	48.5^**a**^±4.3	15.8^**d**^±1.2	27.4^**ab**^±2.0	33.9^**c**^±4.2	2.8^**a**^±0.3
72	pellets	12	9.0^**c**^±5.0 ^x^	39.7^**de**^±4.7	11.8^**d**^±0.8 ^x^	21.2^**c**^±1.5	32.3^**d**^±4.3	2.4^**bc**^±0.4
	straws		33.1^**b**^±12.8 ^y^	41.8^**bc**^±4.0	14.4^**d**^±1.6 ^y^	23.8^**c**^±2.8	36.0^**c**^±2.0	2.5^**a**^±0.2
79	pellets	9	15.7^**c**^±8.7 ^x^	35.8^**e**^±1.3 ^x^	20.2^**a**^±3.0	25.6^**b**^±2.2 ^x^	53.1^**a**^±6.3	1.7^**d**^±0.2
	straws		38.0^**b**^±15.6 ^y^	42.9^**bc**^±2.1 ^y^	22.3^**a**^±3.6	28.6^**a**^±2.8 ^y^	50.4^**a**^±7.8	2.0^**b**^±0.2
Total/Average	**pellets**	**69**	**27.4±16.5 ^x^**	**43.9±7.5**	**16.3±3.3**	**26.1±4.0**	**38.3±8.5**	**2.6±0.7**
	**straws**		**46.3±15.9 ^y^**	**43.1±4.7**	**17.4±3.9**	**26.4±3.2**	**40.9±9.7**	**2.5±0.5**

^a,b,c,d,e^—means in columns for the same freezing method followed by different superscripts differ significantly [P<0.05];

^x,y^—means in columns for the same male indicate significant differences between freezing method [P<0.05].

Comparing the characteristics of thawed semen frozen by the two tested methods, individual differences in the resistance to applied sperm cryopreservation were observed. In relation to live cells in total and sperm motility in semen of six males, higher values (P<0.05) were observed in semen frozen in straws. However, taking into consideration the percentage of live normal sperm, a significant (P<0.05) effect of freezing method was found only for two capercaillies (♂ no. 58 and no. 49) (Table 3). Analyzed motility parameters of thawed semen differed (P<0.05) between males depending on freezing procedure (Table 4).

### Fertilizing ability of thawed semen frozen in straws

Inseminated females laid 32 eggs together and 27 were fertile; the fecundity data of individual capercaillie females are presented in Table 5.

**Table 5:** Fecundity results for capercaillie females inseminated with the thawed semen frozen by straws method (means ± SD).

**Evaluated traits**	**Female 1**	**Female 2**	**Female 3**	**Total/Average**
Number of lied eggs [pcs.]	9	12	11	32
Number of fertile eggs [pcs.]	7	11	9	27
Fertility [%]	77.77	91.66	81.81	84.38
Number of hatched chicks [pcs.]	5	9	6	20
Hatchability of fertile eggs [%]	71.42	81.81	66.67	70.07

## Discussion

It is generally accepted that one important criterion to predict the ability of sperm to withstand freezing and thawing procedures is the quality of the fresh semen [3], [8], [21], [17]. Our earlier experiments showed that quality of capercaillie ejaculates depends on individual male properties and their age [8], [16], but not the way of male management, i.e. keeping them with or without female access [17]. The quantitative and qualitative traits of semen used in this experiment were at a high level, comparable to those presented in our previous publications [8], [22].

The occurrence of species differences in sperm tolerance to the freezing-thawing process [6], [23], [24] requires the development of distinct cryopreservation methods for particular avian species. Such difference are caused by initial cholesterol/phospholipid ratios in the sperm of different species, and additionally the cryopreservation process increases sperm membrane rigidity and in many cases induces a decrease in cholesterol/phospholipid ratio [25]. Spermatozoon membrane permeability, which is not uniform over the entire cell surface, the transport of water occurring mainly through midpiece, is also very important [26], [27].

For rooster semen cryopreservation in pellets, DMA and glycerol are the most often used cryoprotectants; however, the latter one has to be removed before deposition into the oviduct [11], [28], [29]. On the other hand, DMA is not recommended for slow cooling rates in straws because thawed sperm are characterized by low fertilizing potency [7], [12]. Varadi et al. [13] concluded that DMF used for freezing guinea fowl semen in straws and at various cooling rates is not sufficiently effective for this poultry species. Contrary to this, DMF and straw freezing at a temperature decrease rate of 60°C/min is recommended for wild Greylag goose (*Anser anser* L.) [30].

Compared to fresh semen, the cryopreservation methods used in the present study had a negative effect on morphology and motility of frozen-thawed semen. However, this phenomenon is always observed in all animal species. The results obtained show that the straw method allows one to obtain a higher survival rate and number of motile sperm, compared to pellets (60.7 vs. 42.5% and 46.3 vs. 27.4%, respectively). However, 62.9% of cells that withstood the cryopreservation process were damaged morphologically. Finally, the number of live normal sperm and motility parameters were similar in both methods. A similar relationship was observed for guinea fowl semen frozen in straws with 10% ethylene glycol or in pellets with 6% DMA as the cryoprotectant [13]. The survival rate and percentage of live intact cells obtained by the mentioned authors in the pellet method were comparable to our earlier results of an experiment carried out on capercaillie semen [8]. Ciereszko et al. [22], freezing capercaillie semen with the pellet method (using Ovodyl as an extender and 6% DMA), obtained higher (by 14.2%) sperm motility, and better motility parameters (VCL, VSL and ALH). The observed differences within the same species, in number of cells that withstood cryopreservation performed in a similar manner, might result from individual capercaillie resistance to cryoinjury stress, which has already been demonstrated in relation to gander semen [31]. In the fresh semen of two capercaillies, Kowalczyk et al. [8] observed a similar percentage of live normal sperm (61.9 and 61.5%), while the freezing-thawing process withstood 48.8 and 13.8% respectively, with intact morphology. Another interesting observation in this study is individual resistance to cryoinjury stress, depending on the cryopreservation method, including the cryoprotectant used. Equilibration of Japanese quail semen with DMA, compared to DMF, resulted in a higher percentage of live normal cells (26.1 vs. 19.8%), and higher fertility (25.8 vs. 18.2%) [32]. Chalah et al. [28] observed no differences in viability of rooster sperm frozen by the pellet method and 6% DMA or in straws with 6.5% DMF, but fertilizing potency of semen frozen in pellets was higher (88.0 vs. 79.0% of fertile eggs, respectively). For guinea fowl semen, preservation in pellets and 6% DMA was also more efficient (higher survival rate of live, intact sperm) than in straws with 6% DMF [13].

The fertility rate ranging from 77.77 to 91.66% (85.2% on average) obtained after capercaillie female insemination with thawed semen frozen in the straw method can be considered as high and comparable to the rate achieved in our earlier studies (80%), where capercaillies were inseminated with semen cryopreserved by the pellet method [8]. It can be stated that, contrary to other Galliformes species, capercaillie sperm show similar tolerance toward the cryoprotectant added to semen, temperature rate at cooling, and type of cell packaging.

In conclusion, this simple procedure developed for freezing capercaillie semen in straws, without the need for expensive freezing equipment, allows one to obtain high fertilizing potency of thawed sperm, and can be used to create a gene bank of capercaillie semen.
